# Echoes from the Past: A Healthy Baltic Sea Requires More Effort

**DOI:** 10.1007/s13280-013-0477-4

**Published:** 2014-01-12

**Authors:** Aarno T. Kotilainen, Laura Arppe, Slawomir Dobosz, Eystein Jansen, Karoline Kabel, Juha Karhu, Mia M. Kotilainen, Antoon Kuijpers, Bryan C. Lougheed, H. E. Markus Meier, Matthias Moros, Thomas Neumann, Christian Porsche, Niels Poulsen, Peter Rasmussen, Sofia Ribeiro, Bjørg Risebrobakken, Daria Ryabchuk, Semjon Schimanke, Ian Snowball, Mikhail Spiridonov, Joonas J. Virtasalo, Kaarina Weckström, Andrzej Witkowski, Vladimir Zhamoida

**Affiliations:** 1 Geological Survey of Finland, P.O. Box 96, 02151 Espoo, Finland; 2 Finnish Museum of Natural History – LUOMUS, University of Helsinki, P.O. Box 64, 00014 Helsinki, Finland; 3 Palaeoceanography Unit, Faculty of Geosciences, Institute of Marine and Coastal Sciences, University of Szczecin, ul Mickiewicza 18, 70-383 Szczecin, Poland; 4 Uni Climate, Uni Research AS, Allégaten 55, 5007 Bergen, Norway; 5 The Leibniz Institute for Baltic Sea Research, Warnemünde, Seestrasse 15, 18119 Rostock, Germany; 6 Department of Geosciences and Geography, University of Helsinki, P.O. Box 64, 00014 Helsinki, Finland; 7 Department of Stratigraphy, Geological Survey of Denmark and Greenland, Øster Voldgade 10, 1350 Copenhagen, Denmark; 8 Department of Geology, Lund University, Sölvegatan 12, 223 62 Lund, Sweden; 9 Swedish Meteorological and Hydrological Institute, 601 76 Norrköping, Sweden; 10 A. P. Karpinsky Russian Geological Research Institute (VSEGEI), Sredny Prospect 74, 199106 St. Petersburg, Russia; 11 Department of Earth Sciences – Geophysics, Uppsala University, 752 36 Uppsala, Sweden

**Keywords:** Baltic Sea, Climate change, Holocene, Inflow, Multiproxy analyses, Modeling

## Abstract

Integrated sediment multiproxy studies and modeling were used to reconstruct past changes in the Baltic Sea ecosystem. Results of natural changes over the past 6000 years in the Baltic Sea ecosystem suggest that forecasted climate warming might enhance environmental problems of the Baltic Sea. Integrated modeling and sediment proxy studies reveal increased sea surface temperatures and expanded seafloor anoxia (in deep basins) during earlier natural warm climate phases, such as the Medieval Climate Anomaly. Under future IPCC scenarios of global warming, there is likely no improvement of bottom water conditions in the Baltic Sea. Thus, the measures already designed to produce a healthier Baltic Sea are insufficient in the long term. The interactions between climate change and anthropogenic impacts on the Baltic Sea should be considered in management, implementation of policy strategies in the Baltic Sea environmental issues, and adaptation to future climate change.

## Introduction

Increased human activities in marine and coastal areas have altered marine ecosystems worldwide (Jackson et al. 2001; Pandolfi et al. 2003; Halpern et al. 2008, 2012). Such activities include marine traffic, overfishing and pollution, and coastal development. Anthropogenic pressures on the Baltic Sea are also very high because almost 90 million people live in its catchment area. It is a relatively young semi-enclosed inland sea with brackish waters, which make it a sensitive ecosystem.

The environmental problems of the Baltic Sea include, for example, eutrophication, increased chemical pollution, occasional algal blooms, and seafloor hypoxia (HELCOM 2010). During the recent decades, considerable efforts have been made to save and restore the environmental conditions of the Baltic Sea. However, more work is necessary to ensure the health of the sea in the future because it has been hypothesized that ongoing global warming and consequent climate changes may affect the Baltic Sea (BACC Author Team 2008) and amplify the existing environmental problems that the Baltic Sea suffers from.

To be able to provide applicable management recommendations, it is essential to improve our understanding of the natural variability of the Baltic Sea ecosystem and its response to climate and human induced forcing. A deeper scientific knowledge and understanding of the multiple factors that affect long-term changes in the marine environment, and of possible future changes, will provide a basis for improved management and implementation of policy strategies (e.g., the European Marine Strategy Directive) targeted on Baltic Sea environmental issues.

Geologic records of the Baltic Sea, particularly those muddy, organic-rich sediments that have accumulated nearly continuously on the seafloor, provide unique information on past environmental changes. This article presents and discusses the main results of the BONUS INFLOW project, which has used integrated sediment multiproxy studies and modeling to reconstruct past changes in the Baltic Sea ecosystem (e.g., saline water inflow strength, sea surface temperature (SST), seafloor redox conditions, and benthic faunal activity) over the last 6000 years, concentrating on the time period covering the natural climate extremes of the Little Ice Age (LIA), the Medieval Climate Anomaly (MCA) (i.e., Medieval Warm Period), and the Modern Warm Period (MWP). Our aim was to identify the forcing mechanisms of those environmental changes and to provide scenarios of impacts of climate change on the Baltic Sea ecosystem at the end of the twenty-first century ad.

## Study Area

**Fig. 1:**
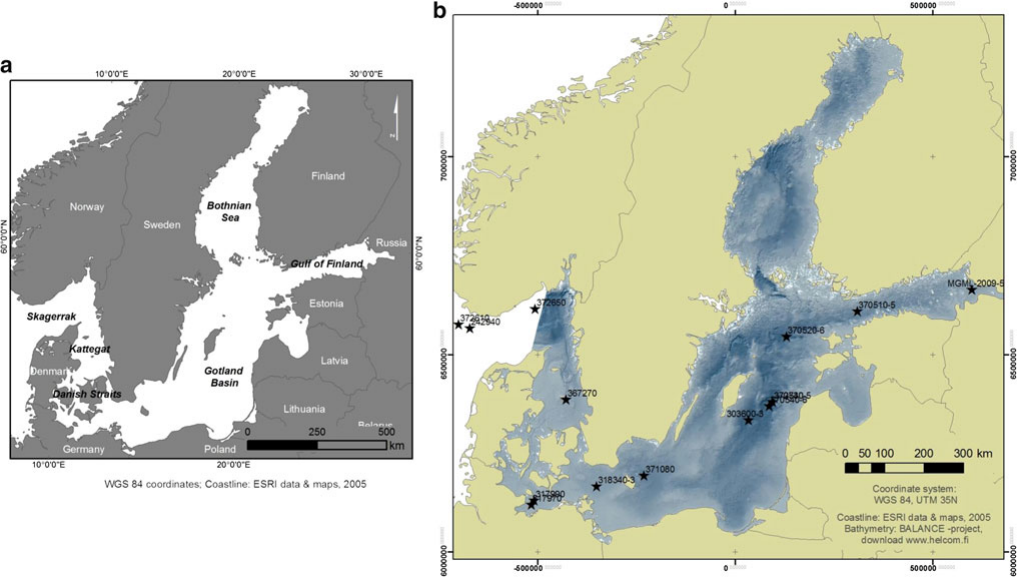
The Baltic Sea in Northern Europe. **a** Subregions of the Baltic Sea. **b** Key sites studied in the BONUS INFLOW project. Sediment core id numbers are shown in the figure. Detailed information on coring locations and water depths are shown in Table 1. The bathymetric map of the Baltic Sea is a product of the BALANCE “Baltic Sea Management – Nature Conservation and Sustainable Development of the Ecosystem through Spatial Planning” Interreg IIIB EU-project (modified from Kotilainen 2012)

The study area covers the whole Baltic Sea, from the marine Skagerrak to the freshwater dominated northern Baltic Sea (Fig. 1). The Baltic Sea is a European inland sea that is one of the world’s largest epicontinental seas and brackish water areas. It is connected to the North Sea through the narrow and shallow Danish Straits. The Baltic Sea covers 415 266 km^2^ with a catchment area four times its size (HELCOM 2013). It is very shallow with an average water depth of ~54 m, although it contains a few large and relatively deep sub-basins (maximum depth of 459 m). The Baltic Sea comprises a series of smaller sub regions (e.g., the Gulf of Finland), each having unique geomorphology (Kaskela et al. 2012) and hydrographic characteristics (Fonselius 1996).

During the last glacial maximum, the entire Baltic Sea basin was covered by an ice sheet (Svendsen et al. 2004); therefore, the history of the present Baltic Sea is geologically very short. The Baltic Sea has developed into its present state during and after the latest deglaciation in the past ca. 16000 years, experiencing both freshwater and marine phases of variable salinities (Björck 1995; Andrén et al. 2000; Emeis et al. 2003). All these past environmental changes can be seen in geologic records, such as the Baltic Sea sediment archives. However, erosion, transportation, and accumulation of the sediments on the seafloor vary spatially and temporally (Winterhalter et al. 1981; Al-Hamdani et al. 2007), resulting in a patchy sediment distribution pattern in the modern Baltic Sea. Thus, selection of sites for sediment proxy studies is crucial. Mud and clay areas (e.g., in basins and plains) include about one-third of the Baltic Sea seafloor (Kaskela et al. 2012). From this part, mud areas represent the areas of relatively continuous sedimentation, as described by Winterhalter et al. (1981), and provide a unique high-resolution archive of the paleoenvironmental history of the large catchment area, the basin itself and the neighboring sea areas.

## Materials and Methods

Several expeditions (e.g., onboard R/V Maria S. Merian, R/V Professor Albrecht Penck, R/V Ladoga, R/V Aranda, and R/V Risk) were organized on the Baltic Sea mainly in 2009 and 2010 to collect material for sediment proxy studies within the Bonus INFLOW project. The key-coring sites (Fig. 1) were selected based on high-resolution topographic information (multibeam echo-sounding data), and shallow seismic surveys, ecosystem modeling, and other available relevant data (such as sediment data from former projects). The sediment material was collected using various techniques. Long sediment cores were recovered using (6 m long) piston corers and (6–9 m long) gravity corers. Short surface sediment cores (< 60 cm) were recovered using mainly a multicorer, and a GEMAX twin-barreled gravity corer.

All sediment cores were digitally imaged, and preliminary lithologic descriptions were prepared onboard. Surface sediment cores and selected long sediment cores were subsampled (mainly) onboard. The surface sediment cores were sliced normally into 0.5- or 1-cm-thick subsamples and packed in plastic bags and boxes. Subsamples of the long sediment cores were taken from selected intervals for various purposes including microfossil (e.g., diatoms, foraminifera, etc.), geochemical, sedimentary fabric, and paleomagnetic/mineral magnetic studies.

Our aim was to study the timing and characteristics of ongoing and past changes in both surface (temperature and salinity) and deep water (oxygen and salinity) conditions. Sediment proxy studies included several methods like TEX86 (a biomarker) for SST (Kabel et al. 2012), strontium isotopes (^87^Sr/^86^Sr) of bivalve shell carbonate and diatoms for salinity, and sedimentary fabric/trace fossil studies for benthic faunal activity reconstructions (Virtasalo et al. 2011a, b). In addition, we used stable isotopes (δ^18^O, δ^13^C), Br (Grigoriev et al. 2011), foraminifera, dinoflagellate cysts, and mineral magnetic analysis. The geochemical methods included XRF scans and ICP-MS analysis.

The key issue for understanding the temporal development of the Baltic Sea based on sediment archives is sound geochronology. Traditional geochronological methods provide results that are too uncertain to achieve sufficiently high temporal resolution. The ^14^C method has been used extensively for dating the Baltic Sea sediments. This method is, however, prone to serious errors due to (1) the scarcity or lack of organic carbon, especially in the early Holocene sediments; (2) the admixing of resuspended older organic material; and (3) the ^14^C deficiency of water (the so-called radiocarbon reservoir effect or marine reservoir effect). The radiocarbon reservoir effect is problematic in the Baltic Sea, its magnitude varies in time and space. Thus, to tackle the radiocarbon reservoir age problem and establish how the reservoir age varied, and to provide the best possible age-depth models for individual core sites, we have applied a range of different techniques. These techniques include ^210^Pb/^137^Cs dating of bulk sediment, AMS-^14^C dating of benthic foraminifera, humic acid and base residue organic carbon fractions of bulk sediment, paleomagnetic dating, and optically stimulated luminescence (OSL) dating. In addition, oceanographic and biological monitoring data were used for identifying further geochronological tie points during the recent past (e.g., footprints of events of major inflow of Atlantic Water to the Baltic in 1993 and 2003 can be seen in the sediment).

Modeling studies were done in close cooperation with sediment proxy studies. The regional climate model of the Rossby Centre (RCA3) was used for downscaling global climate simulations (ECHO-G) to the regional (the Baltic Sea) scale and to deliver lateral boundary conditions for the local ecosystem models (Schimanke et al. 2012). The better constrained ecosystem models (RCO-SCOBI and ERGOM) provided simulated data (hydrographic and biogeochemical conditions) for extreme natural climatic conditions over the past thousand years (e.g., the MCA ~950–1250 ad and the LIA ~1350–1850 ad) (Kabel et al. 2012; Schimanke et al. 2012). These are partly forced with the sediment proxy results such as a 2 °C surface water temperature increase from the LIA toward the MWP. Model experiments provided insight into the mechanisms triggering the Baltic Sea ecosystem state changes as observed in sedimentary archives. Validated models were used for providing scenarios of the Baltic Sea ecosystem state at the end of the twenty-first century for selected Intergovernmental Panel on Climate Change (IPCC) climate change scenarios. Transient simulations for a future climate (1960–2099) were performed using RCAO/ECHAM5-A1B_3, RCAO/ECHAM5-A1B_1, RCAO/ECHAM5-A2, and RCAO/HadCM3-A1B model combinations to provide forcing for the Baltic Sea ecosystem models. Modeling was done in close cooperation with the BONUS ECOSUPPORT project (Meier et al. 2012).

## Key Results and Discussion

### Sediment Material

Nearly one hundred sediment cores (including gravity cores, piston cores, and different types of surface sediment cores) were recovered during the expeditions from numerous carefully selected sites. The key sites (15) are shown in Fig. 1 and listed in Table 1.

**Table 1:** Key sites studied in the INFLOW project. Sea area, sediment core ID number, coordinates (WGS 84), water depth (in m), recovery (the length of sediment core in cm), and research vessel are shown for each site. Additional information is available in Kotilainen et al. (2012b)

Sea area	Core ID	Latitide (N)	Longitude (E)	Water depth (M)	Recovery (cm)	Research vessel
Skagerrak	372610	57°41.05	06°41.00	320	550	Maria S. Merian
Skagerrak	242940	57°40.520	07°10.000	316	890	Poseidon
Skagerrak	372650	58°29.76	09°35.91	550	530	Maria S. Merian
Kattegat	367270	56°41.282	11°46.679	41	379.5	Prof. Albrecht Penck
Mecklenburg Bay	317970	54°12.011	11°21.010	23	758	Maria S. Merian
Mecklenburg Bay	317990	54°18.596	11°25.571	23	865	Maria S. Merian
Arkona Basin	318340-3	54°54.765	13°41.444	47	1104	Maria S. Merian
Bornholm Basin	371080	55°20.37	15°26.76	93	380	Prof. Albrecht Penck
Gotland Basin	303600-3	56°55.01	19°20.01	170	820	Poseidon
Gotland Basin	370530-5	57°23.123	20°15.489	231	498	Aranda
Gotland Basin	370540-6	57°17.011	20°07.248	243	650	Aranda
Northern Central Basin (NCB)	370520-6	58°53.657	20°34.419	182	480	Aranda
Western Gulf of Finland (JML)	370510-5	59°34.907	23°37.572	80	557	Aranda
Eastern Gulf of Finland (F40)	MGML-2009-5	60°06.409	28°47.518	38	454	Aranda

### Sediment Chronology

Besides the improved geochronology, multiproxy dating methods used in the INFLOW project allowed for the inference of radiocarbon reservoir ages. By comparing radiocarbon dates on foraminifera to an independent geochronology based on paleomagnetic secular variation (PSV) and Pb deposition isochrones, a temporal trend toward younger reservoir age values was found (Lougheed et al. 2012). This is possibly due to a temporal reduction in marine water influence due to isostatic uplift of most of the Baltic basin. This agrees with recent spatial reconstructions of contemporary reservoir ages, or *R*(*t*), carried out through ^14^C dating of mollusc shells of known age, whereby a statistically significant relationship between marine water influence and *R*(*t*) was found (Lougheed et al. 2013). Other dating methods tested during the project also included OSL dating. Results indicated complete bleaching and a great promise for the OSL dating technique for marine fine-grained sediments (Kotilainen et al. 2012a).

### Changing SSTs and Anoxia in the Past

The new results of natural past changes in the Baltic Sea ecosystem provide a pessimistic scenario for the future of the sea. Integrated modeling and sediment proxy studies reveal increased SSTs and extended seafloor anoxia (in the deeper basins) also during earlier natural warm climate phases such as the MCA.

**Fig. 2:**
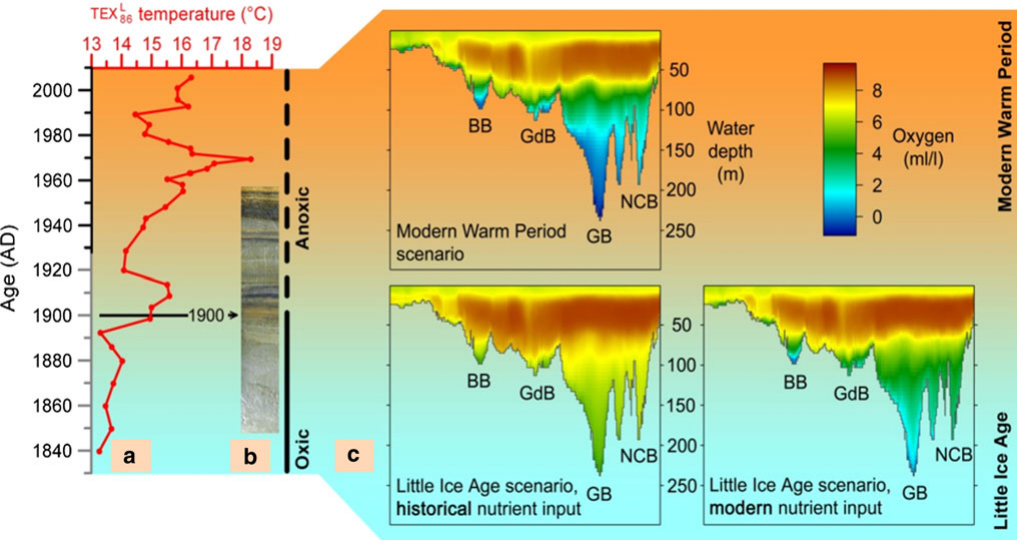
**a** TEX_86_ (biomarker) reconstructed SSTs (°C) (*red curve*) from the Baltic Sea (Gotland Deep) sediment core, over the last 150 years. Anoxic periods at seafloor can be seen in sediment *photograph* (**b**) as laminated structures, and more oxic conditions as homogeneous structures. **c** Oxygen concentrations as simulated with an ecosystem model for the MWP (*upper*), for the LIA with historical (preindustrial) nutrient input (*bottom left*), and for the LIA with modern nutrient input (*bottom right*). Figure is modified after Kabel et al. (2012)

Sea surface temperature reconstructions, based on sediment proxy studies (e.g., the TEX_86_ method), indicate 2–3 °C variability between the MCA, the LIA, and the MWP (Kabel et al. 2012). This variability is higher than expected. Around a thousand years ago, during the MCA, the SST of the Baltic Sea was approximately the same as today (Kabel et al. 2012). This is supported by temperature reconstructions in the shallow water coastal environment of the Kattegat (Kuijpers et al. 2012). Sediment studies reveal that the Medieval Baltic Sea was severely affected by oxygen depletion. During the LIA, the SST of the Baltic Sea was 2–3 °C colder than today, and seafloor oxygen conditions were improved during the LIA. In the Gotland Basin, various parameters recorded oxic conditions during the LIA, and similar LIA results are also reconstructed by ecosystem models (Fig. 2) (Kabel et al. 2012; Schimanke et al. 2012). The establishment of anoxia in the deeper basins began at the same time as the temperature rise from the LIA toward the MWP. In shallower areas, anoxic conditions were established much later. These results highlight a strong effect of SST on redox conditions especially in the central Baltic (Kabel et al. 2012). However, these findings are in contrast to those of the model study by Schimanke et al. (2012). Their experiments suggest that changing nutrient loads may be a more important determinant of oxygen depletion than changes in temperature or dynamic feedbacks. Early human influence on the Baltic Sea ecosystem, like enhanced hypoxia during the MCA, has been proposed by, for example, Zillén et al. (2008) and Zillén and Conley (2010) (but see also Virtasalo 2010). Therefore, further investigations are needed to explain the reconstructed evolution of oxygen concentrations in the Baltic Sea.

### Past Saline Water Inflow Changes, Oxygen Depletion, and Benthic Communities

**Fig. 3:**
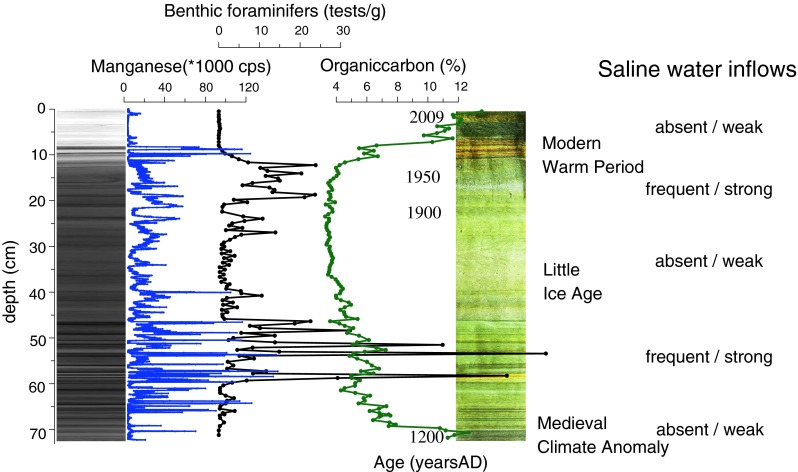
X-ray intensity image of a sediment core from the Gotland Deep, Baltic Sea (*left*); manganese concentration (*blue curve*), number of benthic foraminifera (tests/g) (*black curve*), organic carbon (%) content (*green curve*), and photograph. Also indicated: MWP, LIA, and MCA, as well as the estimated frequency and strength of the saline water inflows. The presence of benthic foraminifers indicate saline water inflows

Sediment records such as benthic foraminifera (mainly *Elphidium excavatum*) counts and XRF scans document another important finding: fewer events of saline water inflows into the Baltic Sea occurred during extended periods of extreme, but stable warm and cold conditions (warm: MWP e.g., 1980–2010, MCA; cold: peak LIA) (Fig. 3). It is probable that saline water inflows increased in frequency and magnitude during climatic transitions. This might be linked to a change in the prevailing atmospheric North Atlantic Oscillation (NAO) system from a stable NAO ± toward more unstable conditions.

Among the new methods used were detailed sedimentologic and ichnologic analyses of sediment X-radiographs. Those were applied, for the first time in the Baltic Sea, for reconstructing faunal community responses to various benthic oxygenation levels, and for identifying basin-scale changes in the composition and functional complexity of zoobenthic communities as a response to changing salinity during the past several millennia (Virtasalo et al. 2011a, b). Furthermore, Fe and S isotope microanalyses of pyrite-filled worm-burrows provided significant new insight into the relative importance of bacterial sulfate reduction and microbial iron reduction in organic carbon mineralization during progressive burial (Virtasalo et al. 2010, 2013).

A significant spin-off from the paleomagnetic (Lougheed et al. 2013) and mineral magnetic (Reinholdsson et al. 2013) studies of the Baltic Sea sediments was the detection of the so-called magnetofossils made of greigite (Fe_3_S_4_), which is a ferrimagnetic mineral that is biosynthesized by magnetotactic bacteria (MTB) for orientation purposes. Sohlenius (1996) reported that sections of laminated Littorina sediments were highly magnetic, probably as a consequence of the precipitation of relatively large grains of authigenic greigite, such as those found in sediments of Yoldia age, but the precise process was not identified. Reinholdsson et al. (2013) were able to show that the magnetic enhancement of laminated Littorina sediments is due to the production and preservation of greigite magnetosomes by MTBs, which is in contrast to the strictly inorganic process that caused greigite to be formed in the older units. Magnetosomal greigite is diagnostic of previous hypoxia and anoxia, and its easily detected presence by geophysical methods provides a rapid means of identifying past occurrences of oxygen deficient conditions, even in unopened sediment cores.

### Future Baltic Sea

Future climate change is likely to affect the Baltic Sea marine environment. Modeling simulations suggest warmer air temperatures in the future in the Baltic Sea region (e.g., Meier et al. 2011). It has been estimated that climate warming could increase precipitation (and river runoff), as well as reduce the length of the ice season in the Baltic Sea. Oxygen depletion on the seafloor has also been estimated to expand (Meier et al. 2011, 2012; Neumann et al. 2012). The changes in hydrography and biogeochemical processes could affect the whole Baltic Sea ecosystem.

Hypoxia is harmful for the macrobenthic fauna and flora. It also affects the ecosystem by facilitating internal loading. Extended and prolonged seafloor anoxia can enhance the environmental problems by releasing toxic heavy metals and nutrients, like phosphorus, from the seafloor sediments, and thus amplify the effects of eutrophication. These may affect the marine ecosystem by reducing biodiversity as well as fish catch. However, reliable future scenarios on the effects of climate change on the Baltic Sea ecosystem and biodiversity are difficult to produce due to complicated “cause–effect” relationships. Further studies are needed.

Socioeconomic implications of climate change on the Baltic Sea region need careful consideration, including effects on fisheries and possible reduced recreational values of the coastal areas. Considerable efforts to save and restore the environmental condition of the Baltic Sea have been made during past decades. However, when combining the effects of climate change, increasing human activities and anthropogenic nutrient loading, the measures already taken are not enough. Further actions, including substantial nutrient load reductions, are needed also in the future to minimize the effect of SST changes.

## Conclusions

The INFLOW project has made significant progress by improving Holocene geochronology of the Baltic Sea, studying past changes in SST and saline water inflows, and integrating sediment proxy and modeling studies.

Integrated sediment proxy and modeling studies have deepened our scientific knowledge and understanding about the factors affecting the past long-term changes and possible future changes of the Baltic Sea environment. The information gained will provide basis for improved management, implementation of policy strategies in the Baltic Sea environmental issues, and for adaptation to the future climate change.

The new results of natural past changes in the Baltic Sea ecosystem, produced by the INFLOW project, provide a pessimistic scenario for the future of the sea. Modeling and sediment proxy results suggest that under the IPCC scenario of a global warming, there is likely no improvement of bottom water conditions in the future (Meier et al. 2011, 2012; Kabel et al. 2012; Neumann et al. 2012). Summing up the effects of climate change, increasing human activities and anthropogenic nutrient loading; nutrient loads, among others, need to be further reduced in the future to minimize the effect of SST changes.

Despite the scientific progress achieved by the INFLOW project some open questions remain. There are still gaps in our knowledge of the mechanisms that link large scale atmospheric forcing to the strong environmental changes that are triggered in the Baltic Sea and observed in sedimentary archives.
